# In this issue: Volume 115, Issue 5, May 2024

**DOI:** 10.1111/cas.16197

**Published:** 2024-05-14

## Cell cycle heterogeneity and plasticity of colorectal cancer stem cells

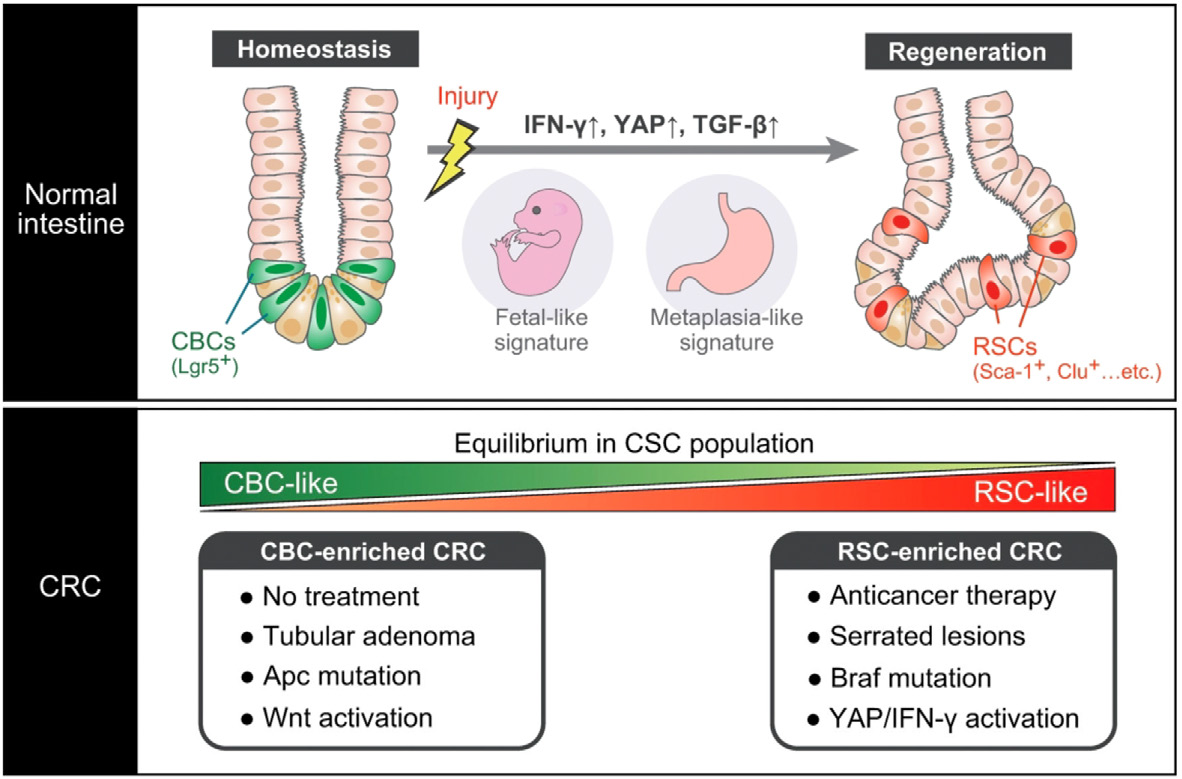

Cancer stem cells (CSCs) are a self‐renewing population of cells found in tumors that drive cancer progression and maintain heterogeneity. Their ability to remain dormant enables them to withstand conventional treatments, resulting in cancer relapse. Colorectal cancer (CRC) poses similar challenges. CRC develops from tubular adenoma or serrated tumors caused by mutations such as *Apc* (adenomatous polyposis coli) gene loss, which activates Wnt and MAPK signaling, suppresses TGF‐β signaling, or *Braf* gene mutation.

Within CRC tumors, quiescent CSCs play a pivotal role, posing challenges to treatment efficacy and contributing to cancer relapse. In a recent review article, researchers explore how CSC populations are sustained in CRC, elucidating the mechanisms of CSC quiescence and the processes underlying CSC pool replenishment.

Colorectal CSCs share similarities with healthy intestinal stem cells (ISCs), including the expression of Lgr5, a receptor for R‐spondin that activates Wnt signaling. These CSCs comprise two subpopulations: actively cycling Lgr5+ CSCs and quiescent Lgr5+ CSCs, with the latter being identifiable using markers such as Mex3a, p27, and p57.

Quiescent CSCs rely on proteins such as p21, p27, and p57 to remain dormant by inhibiting cyclin‐CDK complexes that control cell cycle progression. The collagen protein COL17A1 inhibits focal adhesion kinase (FAK), which maintains p27 expression. Chemotherapy, on the other hand, disrupts this dormancy by activating FAK‐YAP‐TEAD signaling, which temporarily awakens quiescent CSCs to replenish the cycling CSC pool, eventually leading to malignant cell differentiation.

Furthermore, stem cells typically differentiate into multiple cell types, but they can dedifferentiate under certain conditions to replenish the stem cell pool. For example, in response to injury, p57+ enteroendocrine cells can dedifferentiate into stem cells, thereby restoring the intestinal stem cell population. Similarly, in CRC, if CSCs are damaged by chemotherapy, surviving non‐CSC malignant cells can dedifferentiate into CSCs, promoting tumor regrowth.

These processes involve complex reprogramming and transcriptomic changes, such as fetal‐like reversion, in which fetal genes like *Sca‐1* or *Clu* are expressed to generate revival stem cells, and metaplasia‐like conversion, in which p57+ cells dedifferentiate. These mechanisms are critical for replenishing ISCs and persist in CRC, promoting tumor regrowth and contributing to treatment resistance. CRCs originating from tubular adenoma or serrated lesions have distinct relapse mechanisms, highlighting CRC's dynamic adaptability to selective pressures from chemotherapy and subsequent treatment resistance.

https://onlinelibrary.wiley.com/doi/full/10.1111/CAS.16117

## Region‐specific DNA hydroxymethylation along the malignant progression of IDH‐mutant gliomas

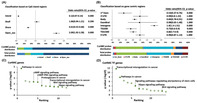

Scientists in the field of cancer research are constantly working to understand the complex processes that lead to cancer development and progression. However, determining the underlying molecular mechanisms of cancer is still a significant challenge. Glioma, a type of aggressive brain tumor, has recently piqued the interest of scientists working to understand the mechanisms underlying its progression. While previous research has shed light on various genetic and epigenetic alterations linked to glioma progression, understanding the role of DNA hydroxymethylation, an epigenetic modification of DNA, in this context is critical.

Motivated by this knowledge gap, researchers set out to investigate the role of DNA hydroxymethylation in the malignant progression of low‐grade isocitrate dehydrogenase‐mutant (IDH^mt^) astrocytoma, a type of glioma known for transitioning from low‐grade to high‐grade tumor. The study shed light on the dynamic nature of epigenetic changes in glioma evolution. To this end, they conducted a genome‐wide hydroxymethylation analysis of these gliomas using advanced techniques such as oxidative bisulfite (OxBS) sequencing and methylation array analysis. The samples used in the study were collected from glioma patients who had undergone tumorectomy between 2001 and 2010.

Their findings revealed significant changes in DNA hydroxymethylation patterns associated with malignant progression, emphasizing the critical role of epigenetic regulation in glioma pathogenesis. Furthermore, they discovered common hydroxymethylated regions in patients, providing invaluable insights into potential cancer‐causing factors. These findings, which shed light on the role of DNA hydroxymethylation in glioma progression, pave the way for the development of novel prognostic markers and therapeutic targets—the identification of critical hydroxymethylated regions opens up avenues for targeted interventions to treat gliomas.

Upon further investigation, they discovered an association between DNA hydroxymethylation alterations and changes in gene expression profiles. Additionally, the presence of KLF4 binding motifs in hydroxymethylated regions suggests a potential regulatory role in IDH^mt^ glioma malignancy.

In conclusion, the study makes a significant contribution to cancer research by providing comprehensive insights into the role of DNA hydroxymethylation in glioma progression. By unraveling the complexities of epigenetic regulation in this context, it takes us a step closer to realizing the full therapeutic potential for glioma patients.

https://onlinelibrary.wiley.com/doi/full/10.1111/CAS.16127

## Clinical features and impact of p53 status on sporadic mismatch repair deficiency and Lynch syndrome in uterine cancer

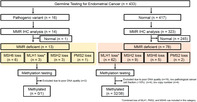

DNA replication is a crucial process responsible for creating copies of DNA strands. Any errors during this process can have deleterious outcomes. The DNA mismatch repair (MMR) system rescues the cell from such incidences by identifying and rectifying unmatched or mismatched DNA.

Lynch syndrome (LS), a genetically inherited condition characterized by pathogenic variants of MMR proteins, compromises DNA mismatch repair. Notably, individuals with pathogenic MMR variants are at an increased risk of developing colorectal, endometrial, and other cancers. However, individuals not carrying these pathogenic variants in their DNA can also develop LS, which is referred to as sporadic LS. Therefore, early screening and diagnosis of LS is crucial. However, there is limited data comparing the prevalence and prognosis of LS and sporadic MMR deficiency (MMRd) in patients with endometrial cancer (EC).

To bridge this gap, Kato et al. investigated the prevalence of LS susceptibility genes in 433 Japanese patients with EC and compared the clinicopathological features and prognosis of MMRd without LS (non‐LS MMRd) and proficient MMR (pMMR). The researchers performed targeted DNA sequencing and identified five LC susceptibility genes, namely: *MLH1*, *MSH2*, *MSH6*, *PMS2*, and *EPCAM*. Next, they assessed the protein status of the MMR variants and the p53 protein using immunohistochemistry (IHC).

The analysis revealed that 3.7% of patients carried pathogenic MMR variants and had LS. Among the 337 patient samples analyzed using IHC, 27% showed the absence of at least one MMR protein. Further, grade 3 endometrioid adenocarcinoma was the dominant histological type in the non‐LS MMRd group, while carcinosarcoma was the dominant tumor in the pMMR group. Notably, 93.8% of the patients with LS and MMR protein loss showed a favorable prognosis with an overall survival rate of 100%. However, there were no significant differences in the survival rate between the LS and sporadic groups. Conversely, non‐LS MMRd patients with aberrant p53 expression had a remarkably worse survival rate compared to those with wildtype p53 tumors (53.6% vs. 93.9%). The results suggest that patients diagnosed with LS typically experience more favorable outcomes and are at a higher risk of developing second cancers than non‐LS patients.

Screening for MMR variants holds prognostic and predictive value in determining the LS susceptibility of patients with EC. Genetic testing can, therefore, help clinicians devise personalized treatment regimes. Moreover, MMR variants and p53 aberrations could be used as potential biomarkers for the molecular classification of EC and risk stratification of patients.

https://onlinelibrary.wiley.com/doi/full/10.1111/CAS.16121

